# The Brazilian Registry of Adult Patient Undergoing Cardiovascular Surgery, the BYPASS Project: Results of the First 1,722 Patients

**DOI:** 10.21470/1678-9741-2017-0053

**Published:** 2017

**Authors:** Walter J. Gomes, Rita Simone Moreira, Alexandre Cabral Zilli, Luiz Carlos Bettiati Jr, Fernando Augusto Marinho dos Santos Figueira, Stephanie Steremberg Pires D'Azevedo, Marcelo José Ferreira Soares, Marcio Pimentel Fernandes, Roberto Vito Ardito, Renata Andrea Barberio Bogdan, Valquíria Pelisser Campagnucci, Diana Nakasako, Renato Abdala Karam Kalil, Clarissa Garcia Rodrigues, Anilton Bezerra Rodrigues Junior, Marcelo Matos Cascudo, Fernando Antibas Atik, Elson Borges Lima, Vinicius José da Silva Nina, Renato Albuquerque Heluy, Lisandro Gonçalves Azeredo, Odilon Silva Henrique Junior, José Teles de Mendonça, Katharina Kelly de Oliveira Gama Silva, Marcelo Pandolfo, José Dantas de Lima Júnior, Renato Max Faria, Jonas Pereira dos Santos, Rodrigo Pereira Paez, Guilherme Henrique Biachi Coelho, Sergio Nunes Pereira, Roberta Senger, Enio Buffolo, Guido Marco Caputi, José Amalth do Espírito Santo, Juliana Aparecida Borges de Oliveira, Otavio Berwanger, Alexandre Biasi Cavalcanti, Fabio B. Jatene

**Affiliations:** 1 Hospital São Paulo - Universidade Federal de São Paulo (UNIFESP/EPM), São Paulo, SP, Brazil.; 2 Hospital de Caridade São Vicente de Paulo, Jundiaí, SP, Brazil.; 3 Instituto de Medicina Integral Professor Fernando Figueira (IMIP), Recife, PE, Brazil.; 4 Hospital de Base - FUNFARME e, São José do Rio Preto, SP, Brazil.; 5 Instituto de Moléstias Cardiovasculares (IMC), São José do Rio Preto, SP, Brazil.; 6 Irmandade da Santa Casa de São Paulo (INCT-HPV/Faculdade de Ciências Médicas da Santa Casa de São Paulo), São Paulo, SP, Brazil.; 7 Instituto de Cardiologia do Rio Grande do Sul - Fundação Universitária de Cardiologia, Porto Alegre, RS, Brazil.; 8 Instituto do Coração de Natal, Natal, RN, Brazil.; 9 Instituto de Cardiologia do Distrito Federal, Brasília, DF, Brazil.; 10 Hospital Universitário da Universidade Federal do Maranhão (HU/UFMA), São Luís, MA, Brazil.; 11 Hospital Evangélico, Cachoeiro de Itapemirim, ES, Brazil.; 12 Hospital do Coração de Sergipe, Aracaju, SE, Brazil.; 13 Instituto de Cirurgia Cardiovascular (ICCV)/Hospital Nossa Senhora da Salete, Cascavel, PR, Brazil.; 14 Hospital Wilson Rosado, Mossoró, RN, Brazil.; 15 Hospital Bosque da Saúde, São Paulo, SP, Brazil.; 16 Hospital Universitário de Santa Maria, Santa Maria, RS, Brazil.; 17 Hospital Universitário de Santa Maria, Santa Maria, RS, Brazil.; 18 Instituto de Pesquisa do Hospital do Coração (IP - HCor), São Paulo, SP, Brazil.; 19 Instituto de Pesquisa do Hospital do Coração (IP - HCor), São Paulo, SP, Brazil.

**Keywords:** Registries, Database, Cardiovascular Surgical Procedures, Cardiac Surgical Procedures

## Abstract

**Objective:**

To report the early results of the BYPASS project - the Brazilian registrY of adult Patient undergoing cArdiovaScular Surgery - a national, observational, prospective, and longitudinal follow-up registry, aiming to chart a profile of patients undergoing cardiovascular surgery in Brazil, assessing the data harvested from the initial 1,722 patients.

**Methods:**

Data collection involved institutions throughout the whole country, comprising 17 centers in 4 regions: Southeast (8), Northeast (5), South (3), and Center-West (1). The study population consists of patients over 18 years of age, and the types of operations recorded were: coronary artery bypass graft (CABG), mitral valve, aortic valve (either conventional or transcatheter), surgical correction of atrial fibrillation, cardiac transplantation, mechanical circulatory support and congenital heart diseases in adults.

**Results:**

83.1% of patients came from the public health system (SUS), 9.6% from the supplemental (private insurance) healthcare systems; and 7.3% from private (out-of -pocket) clinic. Male patients comprised 66%, 30% were diabetics, 46% had dyslipidemia, 28% previously sustained a myocardial infarction, and 9.4% underwent prior cardiovascular surgery. Patients underwent coronary artery bypass surgery were 54.1% and 31.5% to valve surgery, either isolated or combined. The overall postoperative mortality up to the 7^th^ postoperative day was 4%; for CABG was 2.6%, and for valve operations, 4.4%.

**Conclusion:**

This first report outlines the consecution of the Brazilian surgical cardiac database, intended to serve primarily as a tool for providing information for clinical improvement and patient safety and constitute a basis for production of research protocols.

"A journey of a thousand miles begins with one step."

Lao Tzu

**Table:** 

Abbreviations, acronyms & symbols	
AMI	= Acute myocardial infarction
CABG	= Coronary artery bypass grafting
GCP	= Good Clinical Practices
IP-HCor	= Institute of Teaching and Research of Hospital do
	Coração
SBCCV	= Sociedade Brasileira de Cirurgia Cardiovascular/
	Brazilian Society of Cardiovascular Surgery
SUS	= Brazilian public health system

## INTRODUCTION

National Registries of Cardiovascular Surgery represents a valuable tool for assessing and understanding the characteristics of current medical practice. It also comprises a background for improvement in quality, enhance patient safety and determining healthcare changes.

Prominent medical societies throughout the world have established Cardiovascular Surgery National Database, harvesting the benefits provided by the outstanding quality of the collected data^[1,2]^.

In 2010, the Sociedade Brasileira de Cirurgia Cardiovascular/ Brazilian Society of Cardiovascular Surgery (SBCCV) conceived the project of an adult cardiovascular surgery database, which reached full operation in August 2015. In partnership with the Instituto de Pesquisa do Hospital do Coração (IP-HCor), the project was termed BYPASS and the defined dataset encompassed a full range of information necessary for accomplishing the main goal. The project has been solely funded by SBCCV, no external financial source was accepted and is intended to be a continued enterprise^[3,4]^.

Therefore, the aim is to report the early results of the BYPASS project, assessing the data harvested from the initial 1,722 patients included.

## METHODS

### Design

BYPASS - Brazilian registrY of adult Patient undergoing cArdiovaScular Surgery - is a national, observational, prospective, and longitudinal follow-up registry, aiming to chart a profile of patients undergoing cardiovascular surgery in Brazil.

### Setting Up

BYPASS was set-up by SBCCV, and its scientific development and monitoring of data and centers was carried out by the IP-HCor, in a partnership between the two institutions.

### Population and Sites

The participation of cardiovascular sites in the project was voluntary and involved institutions throughout the whole Brazilian territory, in order to obtain a picture of the practice in most regions and states of the federation (Table 1).

**Table 1:** Participating sites and Principal Investigators (PI).

Instituto de Cardiologia do Rio Grande do Sul – Fundação Universitária de Cardiologia – Porto Alegre, RS, Brazil – Renato Abdala Karam Kalil
Hospital Evangélico – Cachoeiro de Itapemirim, ES, Brazil – Lisandro Gonçalves Azeredo
Instituto de Cardiologia do Distrito Federal – Brasília, DF, Brazil – Fernando Antibas Atik
Instituto de Cirurgia Cardiovascular (ICCV)/Hospital Nossa Senhora da Salete – Cascavel, PR, Brazil – Marcelo Pandolfo
Hospital São Vicente de Paulo – Jundiaí, SP, Brazil – Alexandre Cabral Zilli
Instituto de Medicina Integral Professor Fernando Figueira (IMIP) – Recife, PE, Brazil – Fernando Augusto Marinho dos Santos Figueira
Instituto do Coração de Natal – Natal, RN, Brazil – Anilton Bezerra Rodrigues Junior
Hospital Universitário de Santa Maria – Santa Maria, RS, Brazil – Sergio Nunes Pereira
Irmandade da Santa Casa de São Paulo/Faculdade de Ciências Médicas da Santa Casa de São Paulo – São Paulo, SP, Brazil – Valquíria Pelisser Campagnucci
Instituto de Moléstias Cardiovasculares (IMC) – São José do Rio Preto, SP, Brazil – Roberto Vito Ardito
Hospital do Coração de Sergipe – Aracaju, SE, Brazil – José Teles de Mendonça
Hospital São Paulo – Universidade Federal de São Paulo (UNIFESP/EPM) – São Paulo, SP, Brazil – Walter José Gomes
Hospital Universitário da Universidade Federal do Maranhão (HU/UFMA) – São Luís, MA, Brazil – Vinicius José da Silva Nina
Hospital de Base – FUNFARME e FAMERP – São José do Rio Preto, SP, Brazil – Marcelo Jose Ferreira Soares
Hospital do Coração (HCor) – São Paulo, SP, Brazil – Ênio Bufolo
Hospital Wilson Rosado – Mossoró, RN, Brazil – Renato Max Faria
Hospital Bosque da Saúde – São Paulo, SP, Brazil – Rodrigo Pereira Paez

The participating centers were well distributed across the country and located in the regions: Southeast (8), Northeast (5), South (3), and Center-West (1). Currently, 17 centers are formally included and inserting data (Table 1)

The study population consists of patients over 18 years of age, undergoing cardiovascular surgery. The types of operations recorded were: coronary artery bypass graft (CABG), mitral valve, aortic valve (either conventional or transcatheter), surgical correction of atrial fibrillation, cardiac transplantation, mechanical circulatory support (any type, from intra-aortic balloon pump to artificial heart) and congenital heart diseases in adults. The informed consent form was signed for every patient included in the study and the onset of data collection followed the National Clinical Research regulation standards, as well as the Document of the Americas and Good Clinical Practices (GCP) and was approved by the Ethics and Research Committee of the coordinating center and each participating institution.

### Variables

The main variables considered in the dataset, besides demographics, were those related to a greater risk for the patients and for analysis of the primary early, mid-term and late outcomes, when applicable. Part of the set of variables can be seen in Table 2.

**Table 2:** Patient's characteristics and procedural aspects.

Baseline	n/N (%)
Gender (female)	594/1722 (34.5%)
Age; mean ± DP (years)	59.9 ± 12.7 (n=1722)
**Type of patients**	
Public (SUS)	1431/1722 (83.1%)
Insured	166/1722 (9.6%)
Private (out-of-pocket)	125/1722 (7.3%)
Transferred from elsewhere to caring hospital	414/1722 (24%)
**Type of operation**	
Elective	1284/1722 (74.6%)
Urgency	381/1722 (22.1%)
Emergency	54/1722 (3.1%)
Rescue from cath-lab	3/1722 (0.2%)
Procedure recommended by Heart Team	421/1722 (24.4%)
Procedure performed at Hybrid Room	132/1722 (7.7%)
**Preoperative status**	
Stable	1631/1722 (94.7%)
Unstable	77/1722 (4.5%)
Severe	14/1722 (0.8%)
Coronary artery disease (CAD)	1048/1722 (60.9%)
Family history – CAD	516/1722 (30%)
Diabetes mellitus	522/1722 (30.3%)
Dyslipidemia	803/1722 (46.6%)
Hypertension	1268/1722 (73.6%)
Myocardial infarction	489/1722 (28.4%)
ST-elevation myocardial infarction	235/489 (48.1%)
Previous coronary stenting	207/1722 (12%)
Prior cardiovascular surgery	162/1722 (9.4%)
Stroke	78/1722 (4.5%)
Peripheral artery disease	98/1722 (5.7%)
Heart failure	427/1722 (24.8%)
NYHA class I	38/414 (9.2%)
NYHA class II	199/414 (48.1%)
NYHA class III	134/414 (32.4%)
NYHA class IV	43/414 (10.4%)
Renal failure	99/1722 (5.7%)
Dialysis	13/99 (13.1%)
Previous cardiac arrest	58/1722 (3.4%)
Left ventricular ejection fraction < 40%	144/1372 (10.5%)
Active smoker	207/1722 (12%)
Ex-smoker	423/1714 (24.7%)
Definite pacemaker	39/1722 (2.3%)
Chronic obstructive pulmonary disease	136/1722 (7.9%)
Active infective endocarditis	41/1722 (2.4%)
Rheumatic fever	169/1722 (9.8%)
Euro QoL 5D – fulfilled	554/1722 (32.2%)

NYHA = New York Heart Association; QoL = Quality of life; SUS = Brazilian public health system

### Outcomes

The primary outcome of this analysis was cardiovascular mortality during hospitalization, total mortality, and cardiovascular events, such as acute myocardial infarction (AMI), major bleeding, and others.

### Data Collection and Management

Data were collected at the participating centers directly by the surgeon or staffs trained for the administrative work. Quality control and data monitoring was performed by the IP-HCor team, which checked all the insertions into the system, reporting to the center, when necessary, according to the pre-established in the study design.

### Sample Size

In this analysis we report interim analysis of 1,722 patients included until December 31, 2016.

### Statistical Analysis

An exploratory analysis of the data was performed where the quantitative variables were described by mean ± standard deviation, and the qualitative ones were presented in form of absolute and relative frequency. Statistical analyses were performed with the Statistical Package R version 3.3.2.

## RESULTS

A total of 2,331 patients were included in the study by 17 active centers distributed throughout the national territory in the period from August 2014 to December 2016. In this analysis, 1,722 patients were considered, taking into account only those who had the overall data completed until the ultimate follow-up. Regarding origin, 635 patients were from the Southeast region, 480 from the South region, 334 from the Central-West and 273 from the Northeast.

### Participating Centers - Features

Table 1 depicts the BYPASS participating centers across the country and the related principal investigator. From the overall, 83.1% patients came from the public health system (SUS), 9.6% were from the supplemental (private insurance) healthcare systems, and 7.3% from private (out-of-pocket) clinic.

### Socio-demographic and Baseline Characteristics

Table 2 shows the socio-demographic characteristics of the sample and among the 1,722 patients analyzed; the mean age was 59.9±12.7 years. Male patients comprised 66%, 30% were diabetics, 46% had dyslipidemia, 28% previously sustained a myocardial infarction, and 9.4% underwent prior cardiovascular surgery. The most prevalent cardiovascular risk factor was hypertension, reported by 73.6% of the patients included.

### Perioperative Data

Of the 1,722 patients enrolled in this partial analysis, 54.1% underwent coronary artery bypass surgery and 31.5% valve surgery, either isolated or combined. The other procedures performed are listed in Table 3.

**Table 3:** The commonest types of operation.

Procedures	n/N (%)
CABG	840/1722 (48.8%)
Valve	411/1722 (23.9%)
CABG and valve	74/1722 (4.3%)
Aorta	35/1722 (2%)
Adult congenital	28/1722 (1.6%)
Heart transplantation	27/1722 (1.6%)
Aorta and valve	27/1722 (1.6%)
Valve and AF ablation	12/1722 (0.7%)
Aorta, CABG and valve	9/1722 (0.5%)
Valve and adult congenital	5/1722 (0.3%)
Aorta and CABG	4/1722 (0.2%)
CABG and adult congenital	3/1722 (0.2%)
AF ablation and adult congenital	1/1722 (0.1%)
Aorta, valve and AF ablation	1/1722 (0.1%)
CABG, valve and adult congenital	1/1722 (0.1%)
Information under review	211/1722 (12.3%)

AF = atrial fibrillation; CABG = coronary artery bypass graft

### Clinical Outcomes

In relation to complications, stroke was recorded in 1.4% and perioperative myocardial infarction in 0.3%, as can be seen in Table 4. The total intraoperative mortality was 0.3%, and the overall postoperative mortality up to the 7^th^ postoperative day was 4%. The mortality for CABG was 2.6%, and for valve operations was 4.4%, with regard to the 7^th^ postoperative day (Table 5). The overall 30-day mortality was 6.4%.

**Table 4:** Perioperative data and events.

	n/N (%)
**Surgical approach**	
Conventional	1701/1722 (98.8%)
Minimally invasive	21/1722 (1.2%)
Robotic	0/1722 (0%)
Use of cardiopulmonary bypass	1556/1722 (90.4%)
Circulatory arrest	119/1556 (7.6%)
Cardioplegia	1426/1556 (91.6%)
Clinical events	569/1722 (33%)
Myocardial infarction	6/1722 (0.3%)
Major bleeding	140/1722 (8.1%)
Transfusion	481/1722 (27.9%)
Postperfusion syndrome	20/1722 (1.2%)
Arrhythmia	84/1722 (4.9%)
Low cardiac output	80/1722 (4.6%)
Need of vasopressors	794/1722 (46.1%)

**Table 5:** Overall mortality and according to the procedure.

Surgery	Death until 7^th^ postoperative day
CABG	22/840 (2.6%)
Valve	18/411 (4.4%)
Combined	10/155 (6.5%)
Overall	4.0%
	Death until 30th postoperative day
Overall	6.4%

CABG = coronary artery bypass grafting

## DISCUSSION

This report of the Brazilian registrY of adult Patients undergoing cArdiovaScular Surgery (the BYPASS project) encompasses information on the first 1,722 patients undergoing adult cardiovascular surgery in the period from August 2014 to December 2016. The submissions are from 17 hospitals in 10 states across Brazil and the database includes analyses of patient characteristics, the type of surgery, and perioperative outcomes.

With this early set of data, the BYPASS project is taking shape and accomplishing the aim of providing a picture of the Brazilian cardiovascular surgery scenario. Despite several previous attempts to establish a national database, a number of restraint circumstances prevented it to crystallize and become a reality.

Following the successful example set by the STS Adult Cardiac Surgery Database, other national societies instituted similar ventures^[5-8]^. The STS National Database was established in 1989 and initially involved only 4% of the American hospitals undertaking heart surgery. It has grown steadily, and today there are 1,071 cardiac surgery practices taking part in the database, representing more than 90% of all adult cardiac surgery centers across the country^[9]^. Likewise, in the BYPASS project, the institutions participating represent 8% of the Brazilian centers performing adult cardiovascular surgery.

Not surprisingly, CABG surgery makes up the majority of procedures performed across the country, with valve surgery coming next and comprising nearly one-third of all operations. However, this picture displays a current trend, with valve surgery on the rise whereas CABG figure is comparatively steadily abating.

The BYPASS registry is a sort of observational study with the potential of supplying a wealth of information on the practice of cardiovascular surgery in Brazil, and then making possible comparisons and recognizing the differences and the similarities with other countries and continents. Previous reports have addressed the differences and biases in outcomes among Brazil and elsewhere, only hypothesizing about the findings attained^[10]^.

Additionally, from the data obtained, it is possible to rationalize and improve the contemporary optimum management of the patients, aiming to the quality enhancement and patient safety, besides refining the methods and the efficiency leads to reduction of costs and cost-effective procedures, supporting the constantly budget-depleted public and private health system.

The gathered data should serve also to a background to define the best practices and assist in its execution, leading to the standardization of the procedures across the units performing cardiovascular surgery.

The community must bear in mind that the BYPASS registry was established as an initiative and solely funded by the SBCCV, making it an independent and trusted source with no commercial link or financial conflict of interest.

The inclusion of the centers undertaking cardiovascular surgery was voluntary; the list of them provided in Table 1, providing a powerful tool that will enable to generate further analyses of current practice, trends and outcomes in the specialty, bringing reliable information.

A robust adult cardiac surgery database will help to improve quality, enhance research opportunities, and provide an up-todate overview of cardiac surgical activities across the country. The registry provides useful information for government and Health Policy developers, healthcare commissioners and regulators and professional societies.

Two important consequences derive from the data analyses: first, the quality improvement, with performance metrics leading indicators and pointers to changes toward a desirable outcome; second, serving as an authoritative source for clinical research, allowing production and publication of scientific papers and thesis. From now onwards, submission of research protocols is open and very welcomed.

Limitations of the database exist and are mainly related to the full data collection and completeness of the outcomes, with gaps remaining beyond hospital discharge and post procedural 30 days. An audit system is on the way to be implemented to check the accuracy of the gathered information. Additionally, a step forward in outcomes research will be the possible link with the SUS database, strengthening the power of data.

The consecution of this project was made possible by the continuous and strenuous work of the surgeons, centers and personal involved, working voluntarily with willingness and diligence to collect and insert data. We would like to thank all contributors in their support to this very important project, still a work in progress.

Next step involves a breakdown analysis, understanding sub-related data and the aspects of every condition individually studied.

Funding of the project give rise to additional concern, yet to be solved, as it is costly and deals with the constraints of the currently available resources. Future development of this database will depend on the continued support of cardiovascular centers and individual surgeons, also aiming to aggregate further units to increase data quantity and quality.

Therefore, this first report outlines the consecution of the Brazilian surgical cardiac database, intended to serve primarily as a tool for providing information for clinical improvement and patient safety and a basis for production of research protocols.

**Table:** 

Authors' roles & responsibilities
WJG, JAES, JABO, OB, ABC, FBJ Conception and study design; data management; manuscript redaction or critical review of its content; final manuscript approval
Others authors Substantial contributions to the conception or design of the work; or the acquisition, analysis, or interpretation of data for the work; final manuscript approval
