# ERRATUM

**DOI:** 10.21470/1678-9741-2017-0053e

**Published:** 2017

In the article "The Brazilian Registry of Adult Patient Undergoing Cardiovascular Surgery, the BYPASS Project: Results of the First 1,722 Patients", published in the Brazilian Journal of Cardiovascular Surgery 32.2, pages 71-76, the correct name of one of the co-authors is Jonas Gonçalves dos Santos and not Jonas Pereira dos Santos.

